# Near-real time aboveground carbon emissions in Peru

**DOI:** 10.1371/journal.pone.0241418

**Published:** 2020-11-02

**Authors:** Ovidiu Csillik, Gregory P. Asner

**Affiliations:** Center for Global Discovery and Conservation Science, Arizona State University, Tempe, Arizona, United States of America; Kerala University of Fisheries and Ocean Studies, INDIA

## Abstract

Monitoring aboveground carbon stocks and fluxes from tropical deforestation and forest degradation is important for mitigating climate change and improving forest management. However, high temporal and spatial resolution analyses are rare. This study presents the most detailed tracking of aboveground carbon over time, with yearly, quarterly and monthly estimations of emissions using the stock-difference approach and masked by the forest loss layer of Global Forest Watch. We generated high spatial resolution (1-ha) monitoring of aboveground carbon density (ACD) and emissions (ACE) in Peru by incorporating hundreds of thousands of Planet Dove satellite images, Sentinel-1 radar, topography and airborne LiDAR, embedded into a deep learning regression workflow using high-performance computing. Consistent ACD results were obtained for all quarters and months analyzed, with R^2^ values of 0.75–0.78, and root mean square errors (RMSE) between 20.6 and 22.0 Mg C ha^-1^. A total of 7.138 Pg C was estimated for Peru with annual ACE of 20.08 Tg C between the third quarters of 2017 and 2018, respectively, or 23.4% higher than estimates from the FAO Global Forest Resources Assessment. Analyzed quarterly, the spatial evolution of ACE revealed 11.5 Tg C, 6.6 Tg C, 8.6 Tg C, and 10.1 Tg C lost between the third quarters of 2017 and 2018. Moreover, our monthly analysis for the dry season reveals the evolution of ACE at unprecedented temporal detail. We discuss environmental controls over ACE and provide a spatially explicit tool for enhanced forest carbon management at scale.

## Introduction

Tropical forests are an important asset in mitigating climate change by limiting carbon dioxide concentrations in the atmosphere [1]. However, the tropics are a hotspot of global land-use change resulting in deforestation and forest degradation [2], contributing up to 10% of the world’s total annual emissions [3]. To incentivize tropical countries to better manage their forests, the United Nations Framework Convention on Climate Change (UNFCCC) created a mechanism to reduce emissions from deforestation and forest degradation (REDD+) [4]. Countries need to report on their forest carbon stocks and emissions, but the estimates vary greatly between studies due to different data sources and methodologies [5, 6], and countries often under-report their forest losses [7]. There continues to be a need for operational monitoring of tropical forests with increased spatial and temporal detail [8, 9].

Remote sensing technologies are widely used to provide cost-effective solutions for mapping and monitoring aboveground carbon [10]. Using national forest plot networks in tropical countries [11], aboveground carbon density (ACD) is estimated using allometric equations based on field-measured tree characteristics [12]. Airborne Light Detection and Ranging (LiDAR) data may also be used for scaling between field and satellite data [13]. While future space missions will directly estimate the global distribution of forest aboveground biomass [14], current machine learning techniques can utilize passive and active remote sensing products to extrapolate regional measurements over larger areas [10, 15]. Although the spatial resolution usually improves when going from pan-tropical to national-scale estimates of aboveground carbon density (ACD; units Mg C ha^-1^), the spatially explicit temporal evolutions of aboveground carbon stocks and emissions are still poorly known [2, 16].

This study presents an objective and practical high-resolution approach for estimating ACD and aboveground carbon emissions (ACE) at yearly, quarterly, and monthly timesteps and with spatially explicit detail. The emissions are estimated using the stock-difference approach [17], previously used to quantify the aboveground carbon loss and gains over larger time periods [3, 16, 18]. We developed a deep learning framework for the country of Peru at 1-ha resolution by ingesting more than 6 million ha of airborne LiDAR-based estimates of ACD and scaling-up using Planet Dove multi-spectral and Sentinel-1 synthetic aperture radar data, as well as elevation data. Due to its daily revisit frequency, Planet Dove imagery provides sufficient cloud-free spectral data, while Sentinel-1 radar data can penetrate clouds, thus providing up to monthly national-scale coverage of Peru. The final ACE were masked using the Forest Loss layer from the Global Forest Watch datasets [19] to minimize possible artifacts on input datasets over the final ACE estimates. Our mapped ACD and ACE were assessed in conjunction with a series of environmental factors (topography, vegetation, climate). Peru was chosen because it is one of the most biologically diverse regions of Amazonia [20], with high forest cover, moderate deforestation rates, but with trends of increasing deforestation and forest degradation [21]. Our current approach represents a step towards a near-real time monitoring system for tropical aboveground carbon.

## Methods

### Remotely sensed estimators of ACD

Planet Dove satellites were used because of their ability to cover the Earth’s land area daily at a spatial resolution of 3.7 m with four spectral channels: blue (455–515 nm), green (500–590 nm), red (590–670 nm), and near-infrared (780–860 nm) [22]. This high temporal revisit frequency increased the opportunity of capturing cloud-free images and allowed us to generate five quarterly mosaics and three monthly mosaics. These mosaics were created by combining thousands of orthorectified scene products, normalized for sun angle correction and previously processed for top of atmosphere radiance and apparent surface reflectance using atmospheric correction and orthorectification [23]. We used quarterly mosaics in the third and fourth quarters of 2017 (Q3, Q4), and first, second and third quarters of 2018 (Q1, Q2, Q3). The monthly mosaics were created for the months within Q3 2018, namely July, August, and September. To do so, between 64,075 and 101,015 Planet Dove scenes were needed for the quarterly mosaics and between 45,344 and 58,528 for the monthly mosaics (S1 Fig). After mosaicking, residual cloud cover was calculated as the percentage of pixels with clouds after applying Planet’s Unusable Data Mask for each scene considered in the mosaicking process [23]. The residual cloud cover was very low (<4%) except for the quarterly mosaic in the cloudiest quarter, Q1 of 2018, at 15.3% (S1 Fig).

The eight resulting Planet Dove mosaics were generated by transforming the surface reflectance of each Dove scene using a linear fit with co-registered Landsat data from a similar season. This normalization approach aims to minimize scene-to-scene variability, thus enhancing the spatial consistency of the final product. Ultimately, a gradient reconstruction was applied using a seamline removal algorithm to create a long-wavelength adjustment to intensity near boundaries of adjacent scenes, thus obtaining cloud-free, nearly seamless mosaics at 2.34 m spatial resolution (for technical details, see [24]). During the seamline removal, each scene is “flexed” independently to match its neighbor, with values near a scene boundary changing more than values away from a scene boundary [24]. We then derived the Normalized Difference Vegetation Index (NDVI) [25], sensitive to chlorophyll content and sufficiently stable for comparing seasonal changes in vegetation growth [26], and Green Normalized Difference Vegetation Index (GNDVI) [27], useful in separating stressed and senescent vegetation from live green vegetation [28].

The Sentinel-1 mission from Copernicus involves two C-band Synthetic Aperture Radar (SAR) satellites able to penetrate through clouds, thereby complementing optical-based satellite mosaics [10] and revealing forest canopy structural characteristics. We selected the images acquired in interferometric wide swath mode of VH and VV polarizations [29], previously shown as good predictors in ACD estimation [30]. Google Earth Engine (GEE) [31] was used to create two Sentinel-1 VH and VV mosaics (10 m spatial resolution) by taking the median value for each time period of Dove mosaics. The scenes composing a mosaic are available in the GEE, pre-processed for thermal noise removal, radiometric calibration and terrain correction [31, 32]. To account for the influence of topography in the distribution of aboveground carbon, we included Peru-wide elevation information obtained from the Shuttle Radar Topography Mission (SRTM), at 30 m spatial resolution [33]. Topography, among other factors, set fundamental limits on the amount and distribution of aboveground carbon [34]. The blue band from Dove mosaics was removed from the analysis due to its sensitivity to atmospheric artifacts. The rest of biophysical variables (green, red, near-infrared bands, NDVI, GNDVI, Sentinel-1 VH and VV, SRTM elevation) were resampled to 1 ha resolution, the official reporting unit for carbon stocks. From a modeling perspective, elevation and spatial information are controlling the spatial distribution of aboveground carbon, while Planet Dove and Sentinel-1 features provide information related to vegetation condition and growth, canopy structure and forest disturbances.

### Airborne LiDAR canopy heights and ACD estimates

The airborne LiDAR data were acquired during two flight campaigns in 2011 and 2013 with the Global Airborne Observatory (GAO; formerly Carnegie Airborne Observatory, CAO), using the Airborne Taxonomic Mapping System (AToMS) instruments onboard the aircraft [35]. Using a national stratification based on geologic substrate, soils, topography and shifts in community composition, GAO acquired 278 trillion precisely georeferenced LiDAR points. An elaborate workflow was deployed in order to extract highly accurate first and last returns from the LiDAR points, which were further used to obtain a digital terrain model (DTM) and a digital surface model (DSM) by taking the last and first returns, respectively [34]. By subtracting the DTM from the DSM, they obtained top-of-canopy height (TCH) measurements at 1.1 m for 6,176,586 ha across Peru, which were resampled to 1 ha grid cells overlapping the rest of remotely sensed data used in this study. TCH alone is highly correlated with ACD, so LiDAR-based ACD estimates were derived using an equation proposed by Asner et al. [34], based on correlating 272 field-based estimates of ACD with TCH (Eq 1), which resulted in a mean error of 11.6%.

$$ACD=0.8245\times TCH^{1.573}$$ (1)

### Deep learning estimation and validation of ACD

Deep learning neural networks have become popular tools to estimate aboveground forest biomass [36, 37] or ACD [30, 38], having a powerful capacity to learn from large and complex data using neurons to link input features to a response variable [39]. The model hyper-parameters were iteratively tuned to create a wide and deep neural network architecture using five layers, with three hidden layers of 250 neurons each. A rectified linear unit activation function for the hidden layers and a linear activation function for the output layer ensured the capabilities of the model to learn non-linear complex relationships between the input features and response variable. The mean absolute error was used as a loss function with an Adam optimizer [40]. Except for the response variable (LiDAR-based ACD), all other environmental features were normalized to a common range of values (0–1) because neural networks are sensitive to this issue. Beyond the Dove, Sentinel-1 and SRTM elevation features, we also included in the neural network model the spatial location (x, y positions) because this was shown to outperform machine learning regression models without spatial contextual modeling for ACD estimation [41].

The same architecture of the model was applied for each period analyzed, with Planet Dove, Sentinel-1, elevation and spatial context as input features to upscale airborne LiDAR ACD estimates. The LiDAR-based ACD estimates were split into 80% training samples and 20% validation samples. The training samples were split again into 80% training and 20% validation samples to create five deep learning estimates that were ultimately averaged into a wall-to-wall Peru map of ACD estimates for each period analyzed. The remaining 20% of samples were not used for training the model and were solely used to validate the final ACD estimates.

The uncertainty of our ACD estimates was evaluated by creating continuous nationwide spatially explicit maps for each 1-ha mapping unit with absolute uncertainty (root mean square error, RMSE) and relative uncertainty (RMSE percentage of estimated ACD). These maps were obtained by binning the estimated ACD values in 10 bins, calculating the RMSE for each bin and fitting polynomial and logarithmic functions for absolute and relative uncertainty, respectively. These types of functions were those who gave the highest R^2^ with significant p value. When applied for each 1-ha pixel, these fitted functions led to maps with continuous variation of uncertainty throughout Peru. Besides this first type of uncertainty, we also considered the 11.6% error in correlating field-based estimates with airborne LiDAR TCH [34] which was combined with our relative uncertainty by computing the square root of the two squared errors.

### ACE: Aboveground carbon emissions

From the quarterly and monthly deep learning estimates of ACD, we created estimates of ACE by subtracting Q3s of 2017 and 2018 (yearly emissions), each consecutive quarter (quarterly emissions) and each consecutive month (monthly emissions) (S2 Fig). To protect the final ACE from artifacts propagated by the input datasets (spectral and radiometric artifacts, cloud coverage), the emissions were masked using the Forest Loss layer, part of the Global Forest Watch datasets [19]. Since the Forest Loss layer is aggregated yearly, the corresponding time period for each ACE estimate was used (2017, 2018 or both aggregated).

## Results

### ACD estimation and uncertainty

**Fig 1:**
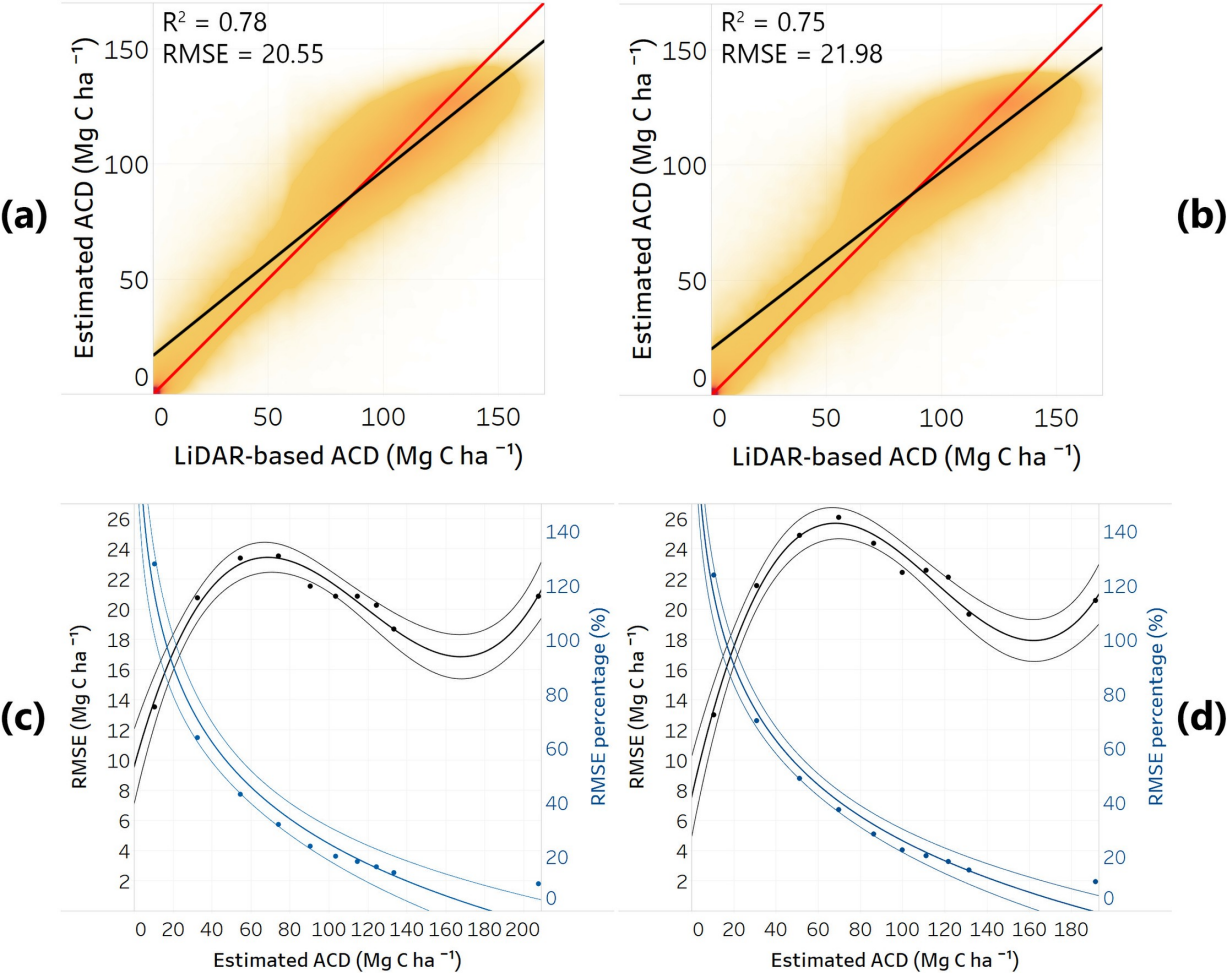
Density scatter plots between our deep learning model-estimated ACD against 20% hold-out airborne LiDAR-based ACD (Mg C ha^-1^). Here are shown **(a)** the highest accurate model, Q3 of 2017 and **(b)** the least accurate model, Q1 of 2018. The 1:1 line is shown with red and the trend line is shown with black. R^2^ and RMSE (Mg C ha^-1^) values are shown in the upper left part. In **(c)** and **(d)** are shown the absolute RMSE (Mg C ha^-1^, black) and relative RMSE (%, blue) values from the estimated ACD for the two models shown in **(a)** and **(b)**, respectively. These uncertainties were obtained by grouping the RMSE values in 10 bins and fitting polynomial and logarithmic functions for the absolute and relative RMSE, respectively. Applied on each 1-ha pixel, these fitted functions gave maps of continuous variation of ACD estimation uncertainty. Each trend line depicted represents the fitted function with confidence intervals.

Nationwide, model-based ACD estimates for all quarters had R^2^ values of 0.75–0.78 relative to airborne LiDAR-based ACD, with the lowest value for the cloudiest quarter (Q1 2018) and higher values for drier quarters (Fig 1). The root mean square error (RMSE) for each quarter followed the same pattern, with values ranging from 20.6 to 22.0 Mg C ha^-1^ (Table 1). All monthly analyses had R^2^ of 0.78 and RMSE values around 20.7 Mg C ha^-1^. These statistics were calculated after validating the results against the 20% (988,167 ha) of LiDAR-based ACD estimates, held out from training the deep learning models.

**Table 1:** Model-based ACD validation for all periods analyzed, showing the mean absolute error (MAE, Mg C ha^-1^), root mean square error (RMSE, Mg C ha^-1^) and R^2^.

*Year*	*Period*	*MAE*	*RMSE*	*R*^*2*^
2017	Q3	14.31	20.55	0.78
	Q4	14.74	21.03	0.77
2018	Q1	15.49	21.98	0.75
	Q2	14.48	20.76	0.78
	Q3	14.56	20.82	0.78
	Jul	14.40	20.66	0.78
	Aug	14.51	20.79	0.78
	Sep	14.52	20.77	0.78

Generating spatially explicit errors for each 1-ha pixel of Peru resulted in a consistent pattern for RMSE across all five quarters and three months analyzed (Fig 1). The absolute RMSE values increased up to 23–25 Mg C ha^-1^ for estimated ACD values around 70 Mg C ha^-1^, with higher RMSE for cloudier quarters. The RMSE values then decreased to 18 Mg C ha^-1^ for high estimated ACD values (100–150 Mg C ha^-1^). For very high estimated ACD values (>150 Mg C ha-1) the RMSE values increased due to the saturation of input variables to higher biomass values and reduced representativeness of the LiDAR samples for these extreme ACD values (Fig 1).

When the absolute RMSE value for each pixel was transformed into %RMSE of estimated ACD, the logarithmic function fitted showed a decline in the relative RMSE with the increase of estimated ACD values (Fig 1). Estimated ACD values higher than 100 Mg C ha^-1^ had declining relative RMSE from 20% down to 10% (Fig 1). These small relative RMSE for high ACD is desirable when mapping ACD, since the majority of the carbon is stored in tall and large trees.

### Large-scale ACD and ACE

**Fig 2:**
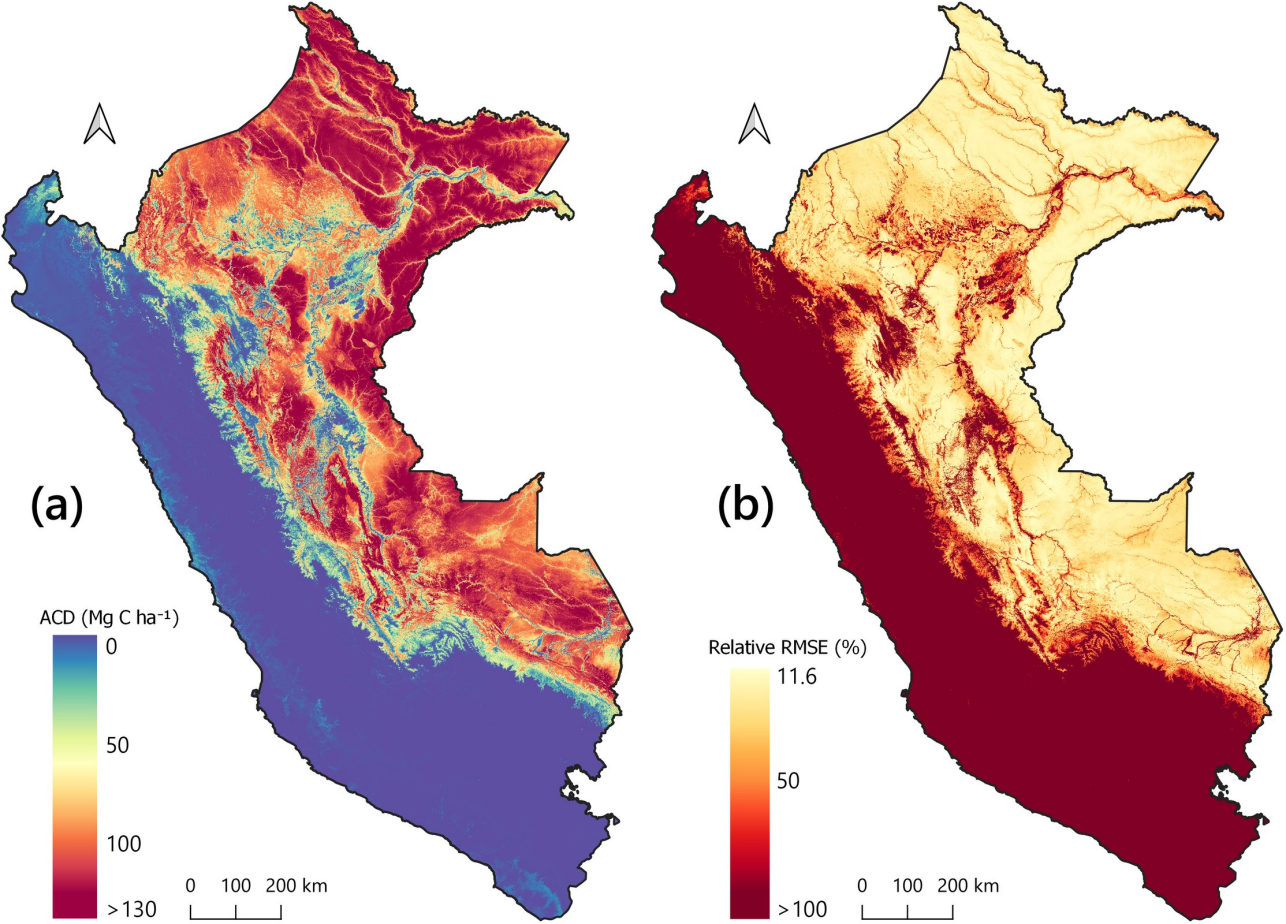
The country-wide ACD estimation and associated uncertainty. Shown here are the **(a)** ACD (Mg C ha^-1^) for Q3 2018, the most recent period analyzed and its **(b)** associated combined relative uncertainty (% RMSE).

The nationwide spatial distribution of ACD was highly influenced by environmental conditions (Fig 2). Lowland Amazonian rainforest was the highest aboveground carbon storage ecosystem, with values exceeding 150 Mg C ha^-1^, while the high Andes and drier coastal regions were relatively low in aboveground carbon storage (Fig 2). The combined uncertainties resulted from our estimation of ACD and the calibration of field-estimated ACD with airborne LiDAR TCH measurements followed the same environmental gradients, with uncertainties for each 1-ha pixel lower than 20% for ACD values higher than 100 Mg C ha^-1^ (Fig 2).

**Fig 3:**
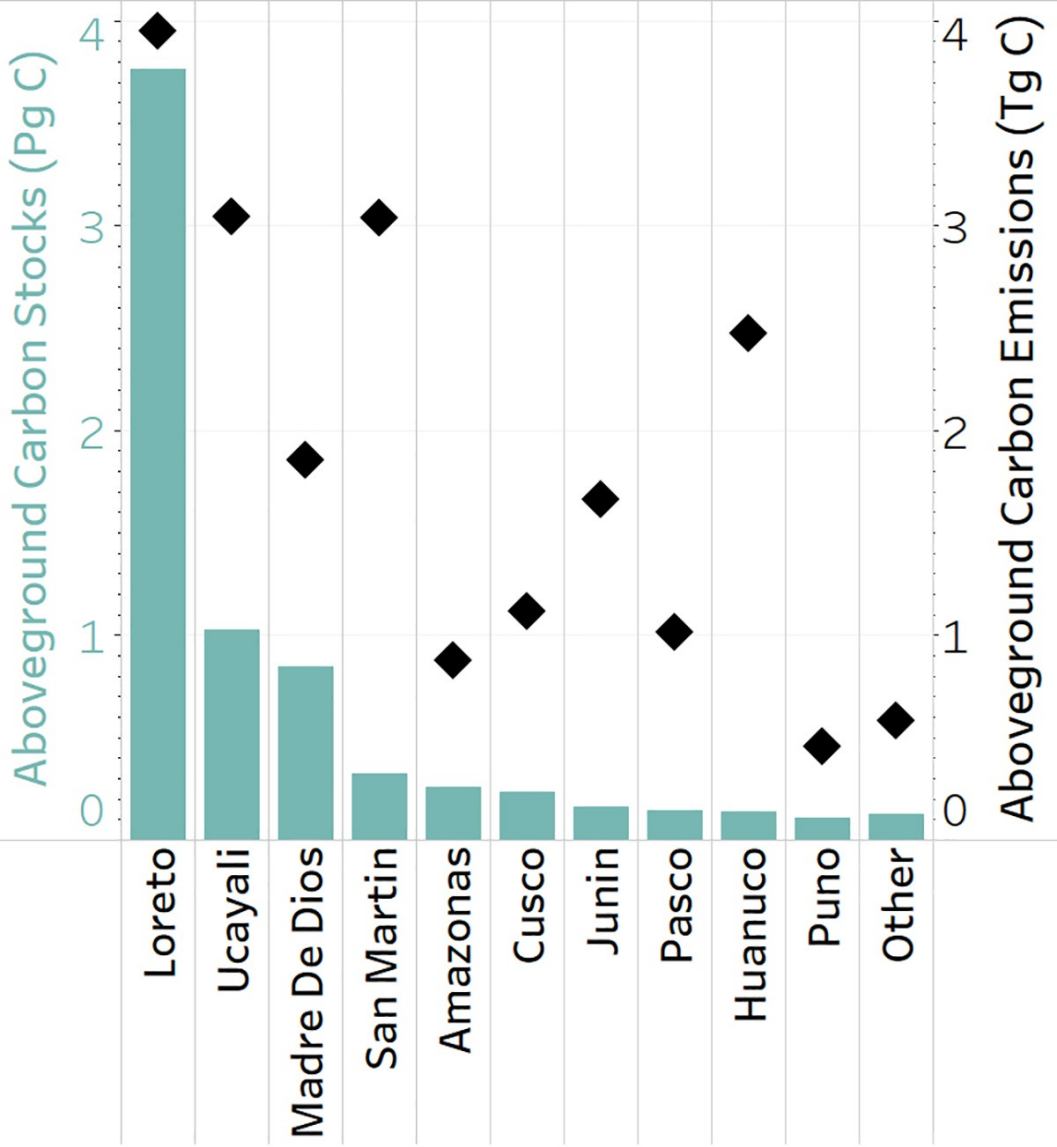
Regional aboveground carbon stocks and emissions statistics. Aboveground carbon stocks for the most recent period analyzed, Q3 of 2018 (Pg C, blue bars), in relation to the yearly ACE (Tg C, black diamonds).

The yearly ACE (Tg C) for Peru totaled 20.08 Tg C. Among the 10 legal regions harboring most of the aboveground carbon in Peru, Loreto (3.95 Tg C), Ucayali (3.04 Tg C) and San Martin (3.04 Tg C) were the leading regions for annual ACE, followed by Huanuco (2.47 Tg C), Madre de Dios (1.86 Tg C) and Junin (1.66 Tg C) (Fig 3). However, when ACE were weighted by the total amount of carbon stored for each region, then Huanuco (1.79%), Junin (1.01%), San Martin (0.94%) and Pasco (0.71%) were the top emitters with alarming rates for a single year. On the other side, Loreto (0.10%), Madre de Dios (0.22%), and Ucayali (0.29%) had lower percentages of ACE in relation to the total amount of carbon stored, due to their larger extent and mostly comprising the Amazonian rainforest.

**Fig 4:**
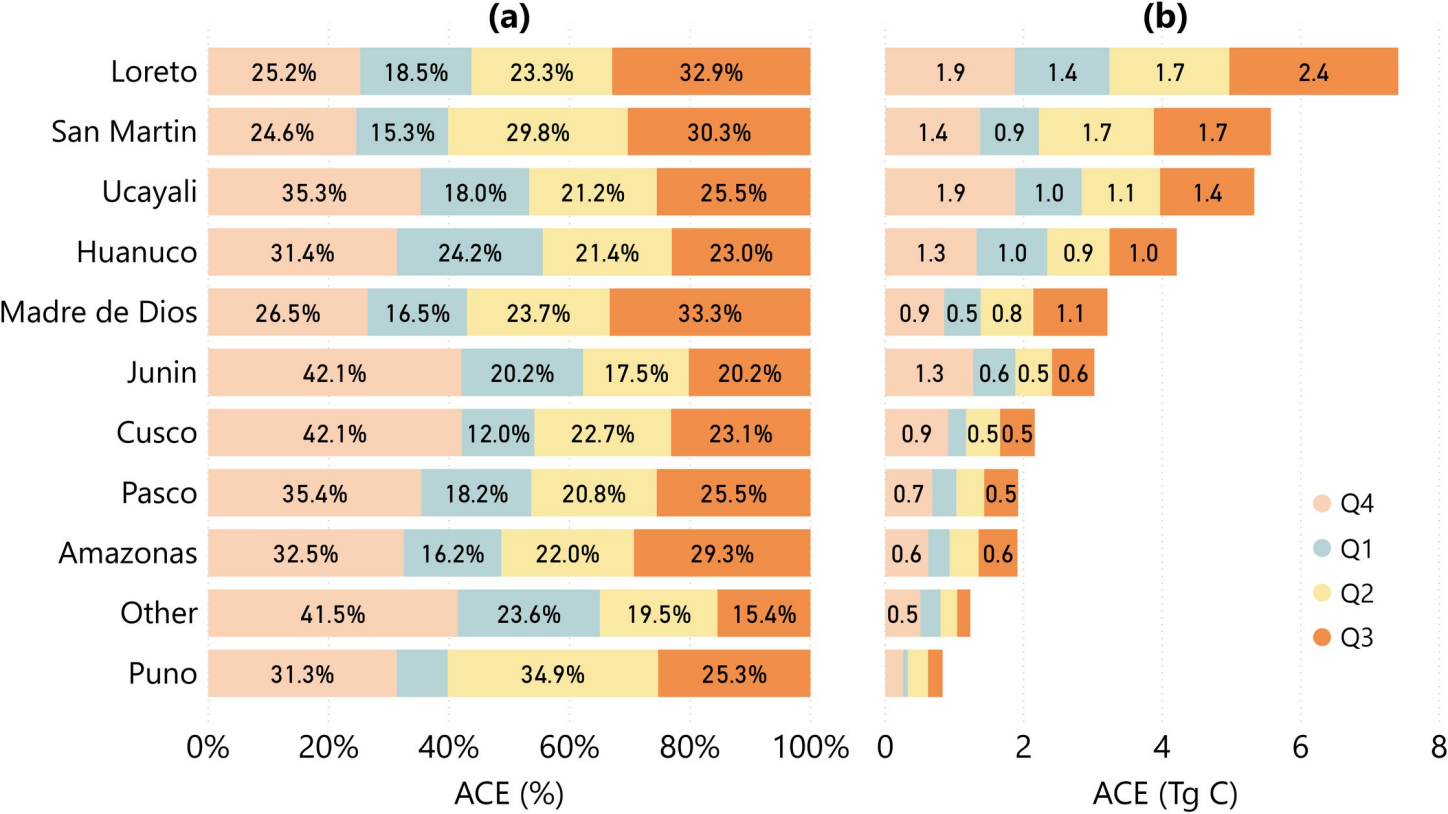
Quarterly ACE statistics at regional level. **(a)** Yearly distribution of quarterly ACE statistics for all legal regions in Peru and **(b)** the quarterly ACE values for these regions (Tg C), between Q4 of 2017 and Q3 of 2018.

The pattern of quarterly ACE between the dry seasons of 2017 and 2018 was influenced by climatic conditions, with most ACE occurring in or around the dry season (from Q3 to Q4 in 2017 and from Q2 to Q3 in 2018) (Fig 4). Lowest quarterly ACE was in Q1 of 2018, the cloudiest quarter analyzed. The decline in ACE from Q4 of 2017 to Q1 of 2018 reached 73.1% for Puno, 71.4% for Cusco, 52.0% for Junin and 50.0% for Amazonas (Fig 4). The quarterly ACE trend increased back towards the Q3 of 2018, with Loreto (2.44 Tg C), San Martin (1.69 Tg C), Ucayali (1.36 Tg C), and Madre de Dios (1.07 Tg C) emitting more than 1 Tg C. This regional trend for quarterly ACE is maintained at the national level, with 11.54 Tg C (31.36% of yearly ACE) emitted in Q4 of 2017, 6.62 Tg C (17.99%) in Q1, 8.55 Tg C (23.23%) in Q2, and 10.09 Tg C (27.42%) in Q3 of 2018.

Monthly ACE totaled 6.93 Tg C in August and 6.57 Tg C in September 2018, with Loreto, San Martin, Ucayali, Juanuco, and Madre de Dios as leading regions. Transitioning from yearly to quarterly and then to monthly ACE might have overestimated the total ACE due to the masking of the final results using yearly forest loss masks.

### Environmental controls on ACE distribution

The distribution of ACE was analyzed in conjunction with climatic and topographic conditions throughout Peru. The yearly ACE patterns in relation to the four environmental factors were similar to those of the ACD distribution. Therefore, the higher the mean annual temperature, the higher the ACE, with a maximum of 7.4 Tg C emitted for the interval of 26–27°C. Relative to annual precipitation, most of the ACE happened in regions with 1400–2400 mm. At lower elevation and on shallow slopes, ACE was higher, with 9.6 Tg C emitted only for elevation between 100–300 m.

**Fig 5:**
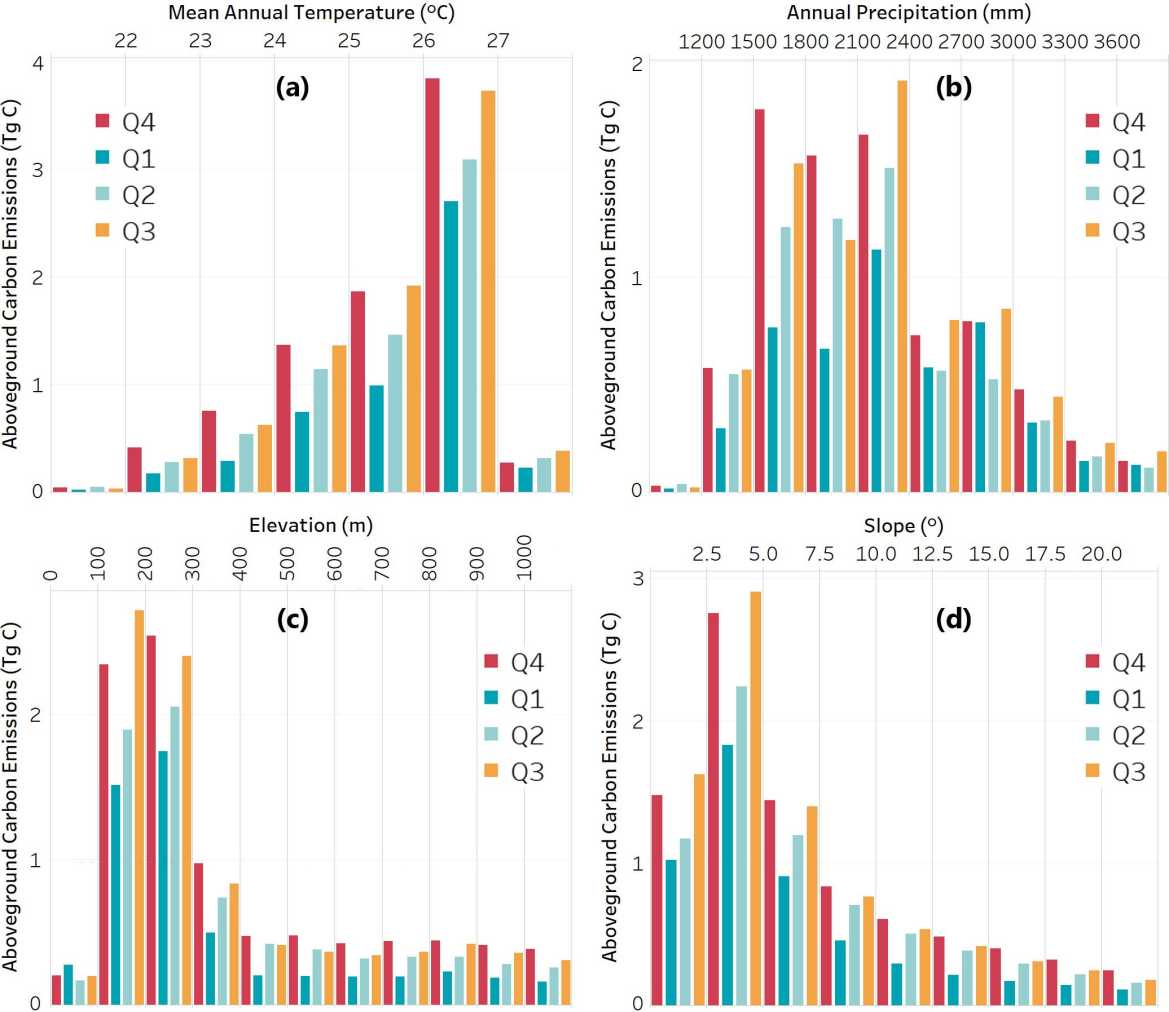
Environmental controls on ACE distribution. Quarterly ACE in relation to the **(a)** mean annual temperature, **(b)** annual precipitation, **(c)** elevation, and **(d)** slope.

When analyzing quarterly ACE, the pattern for all four environmental variables was similar, with the highest emissions in Q4 of 2017 and Q3 of 2018. Lower ACE occurred in Q1 and Q2 of 2018 (Fig 5). Interestingly, the quarterly distribution of ACE between 0 and 100 m was not following this trend, having Q1 emissions the highest (Fig 5C). This was attributable to the meandering of the rivers (non-anthropogenic deforestation) in the lowland regions of Ucayali and Loreto, which happened in the rainy season overlapping Q1 and Q2.

### Aboveground carbon protection and threats

From 7.138 Pg C of Peru’s total aboveground forest carbon, 3.042 Pg C occurred in protected areas. National parks (1.015 Pg C) and buffer areas (1.079 Pg C) stored most of this carbon in lowland Amazonia and the Andean highlands. Other forms of protection, like national reserves, regional conservation areas or communal reserves, stored 0.948 Pg C. Different levels of protection and the wide range of enforcement levels were reflected in annual ACE, which were low for national parks and reserves (0.23 and 0.15 Tg C respectively), but very high for their buffer areas, with 4.77 Tg C. In general, the quarterly ACE were higher towards the dry season and lower otherwise, but there was a high variability on the quarterly distribution of ACE for protected areas.

**Fig 6:**
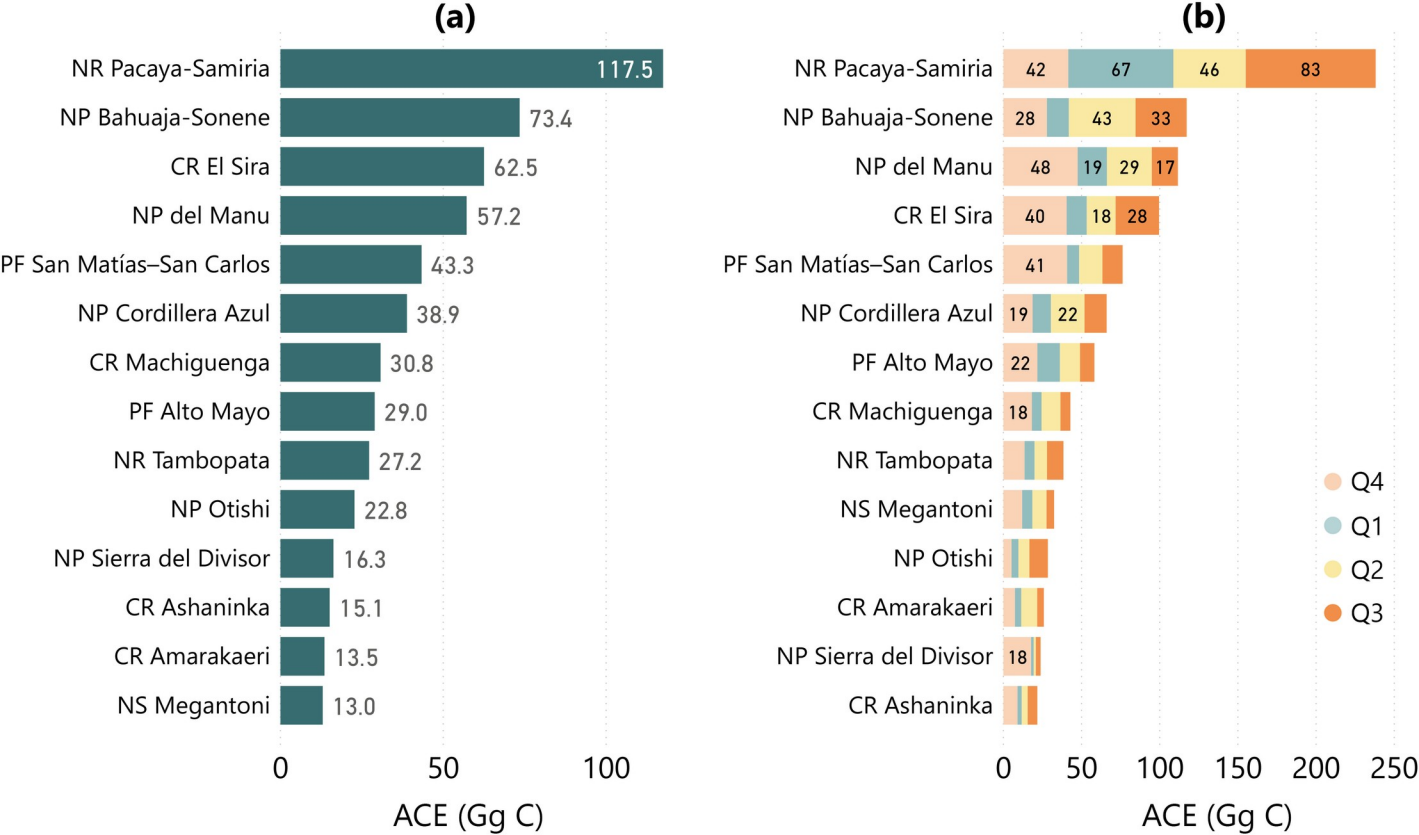
National protected areas with (a) high annual ACE (in Gg C) and (b) the quarterly ACE results for these areas (in Gg C). Abbreviations: National Park (NP), National Reserve (NR), Communal Reserve (CR), Protection Forest (PF), National Sanctuary (NS). Note that 1,000 Gg C = 1 Tg C.

**Fig 7:**
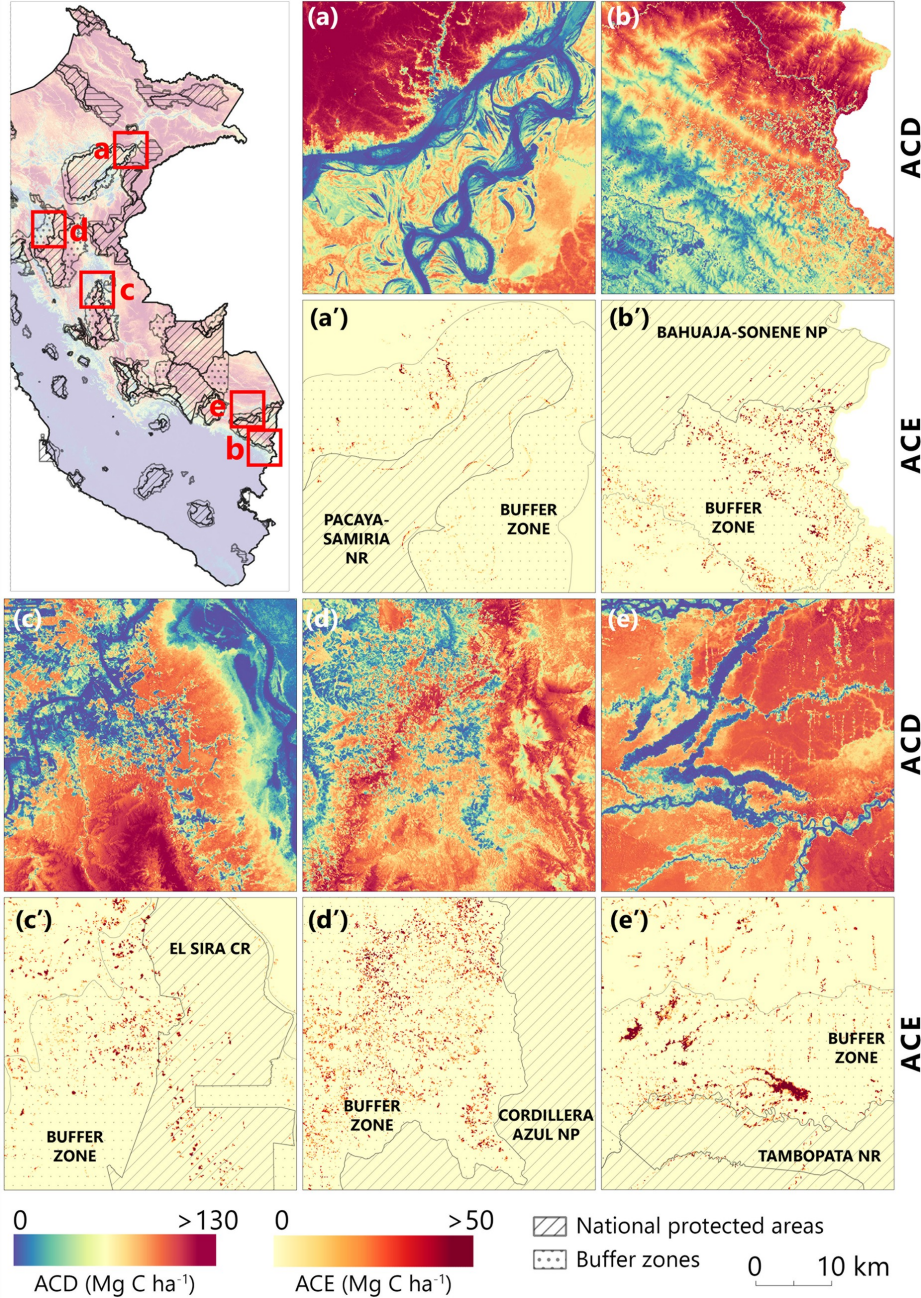
Five examples of ACD distribution in 2018’s Q3 and annual ACE for protected areas and their buffer zones. The location of each subset is depicted on the map of Peru (upper left), with **(a)** Pacaya-Samiria NP, **(b)** Bahuaja-Sonene NP, **(c)** El Sira CR, **(d)** Cordillera Azul NP, and **(e)** Tambopata NR showing examples of forest disturbance entering the protected areas (high level of enforcement) and highly affecting their buffer zones (low level of enforcement, multiple uses).

Four protected areas had annual ACE higher than 50 Gg C, with diverse factors leading to emissions (Fig 6). The National Park Pacaya-Samiria overlaps large portions of swamp forest in the region of Loreto, but the triggering factor for the 117.5 Gg C emissions was the intense meandering of the rivers during and after the rainy season (Fig 7A). This was visible on the spatially explicit maps and statistics of quarterly analysis that had high values of ACE in Q1, Q2, and Q3 of 2018 (Fig 6). Bahuaja-Sonene National Park (73.4 Gg C annual ACE) was threatened by the expansion of gold mining activities in the Madre de Dios region, as well as deforestation and forest degradation happened at the border with Bolivia (Fig 7B). The Communal Reserve El Sira, at the intersection of Ucayali, Huanuco, and Pasco regions, was highly exposed by the urban and agricultural expansions south of the city of Pucallpa (Fig 7C). Last but not least, National Park Del Manu recorded 57.2 Gg C of annual ACE caused by spurious forest degradation along the main rivers.

**Fig 8:**
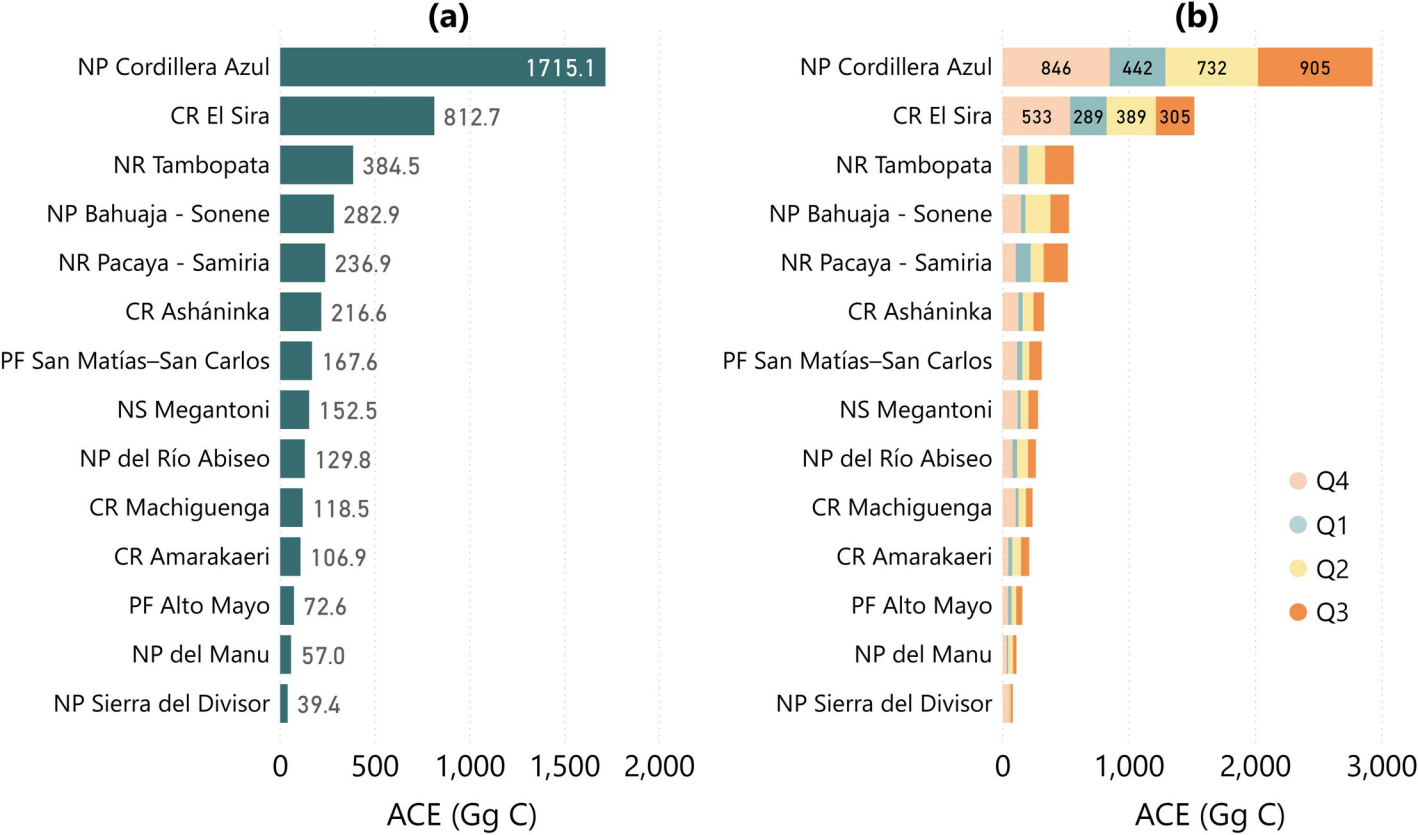
Buffer zones of national protected areas with (a) high annual ACE (in Gg C) and (b) the quarterly ACE results for these areas (in Gg C). Abbreviations: National Park (NP), National Reserve (NR), Communal Reserve (CR), Protection Forest (PF), National Sanctuary (NS). Note that 1,000 Gg C = 1 Tg C.

The buffer zones had multiple uses and lower levels of enforcement and were exposed to a multitude of threats (Fig 8). The buffer zone for the National Park Cordillera Azul recorded the highest annual ACE, 1715 Gg C, with high degradation areas, especially south of the city of Tarapoto (Fig 7D). The buffer zone of Communal Reserve El Sira emitted 812 Gg C from intense deforestation south of the city of Pucallpa (Fig 7C), while the buffer zone of National Reserve Pacaya-Samiria (236 Gg C annual ACE) was affected by intense river meandering and deforestation along the Iquitos-Nauta road (Fig 7A). The buffer zones of National Reserve Tambopata (384 Gg C) and National Park Bahuaja-Sonene (0282 Gg C) in southeastern Peru had large areas affected by illegal gold mining, deforestation along the Interoceanic Highway or along the border with Bolivia (Figs 7E and 8).

## Discussion

We generated and presented a high spatial and temporal resolution analysis of ACE for Peru by using a combination of airborne LiDAR, Planet Dove images and Sentinel-1 radar data within a deep learning model framework. To our knowledge, this is to-date the most detailed large-scale aboveground carbon mapping analysis through time, and provides a new contribution to our understanding of forest carbon dynamics in the region. Previous studies have developed modeling approaches of carbon emissions for the pantropical level using remote sensing data [16] or in combination with a carbon bookkeeping model [3], but with lower spatial and temporal resolution. Spatially explicit ACE were derived by using the stock-difference method in line with the IPCC guidelines [17] at a land management scale (1 ha), thereby directly supporting mitigation and reporting by land managers and decision-makers.

The temporally detailed aspect of our study was achieved by using daily Planet Dove images and incorporating them into monthly and quarterly mosaics. Planet Dove images have previously been used for different land cover applications [42, 43] and more recently for estimating ACD [18]. Computational advances allowed us to use more than 100,000 high-resolution Dove scenes for a single mosaic that ensured the least amount of cloud cover after cloud masking. Even so, creating monthly mosaics for the wettest periods of the year proved challenging and only feasible for the drier months. This would have not been possible with other optical sensors used in estimating biomass or carbon stocks, like Sentinel-2 [44], Landsat-8 [34, 45, 46] or MODIS. In addition, the Sentinel-1’s ability to penetrate the clouds was demonstrated in the past for forest monitoring [47, 48] and was proven a good addition to the spectral information of Planet Dove imagery. Our approach is flexible and Sentinel-2 or Landsat-8 are viable alternatives to Planet imagery, with higher spectral resolution, but lower spatial and temporal resolutions. Higher spectral resolution would further increase the accuracy of ACD estimates, as was demonstrated using shortwave infrared spectral bands from Landsat, sensitive to characteristics of closed canopy forests [46], or red-edge spectral bands from Sentinel-2 [49]. The new generation of Planet Super Dove satellites now acquire images with eight spectral bands, and will eventually offer daily high spatial and spectral resolution coverage.

**Fig 9:**
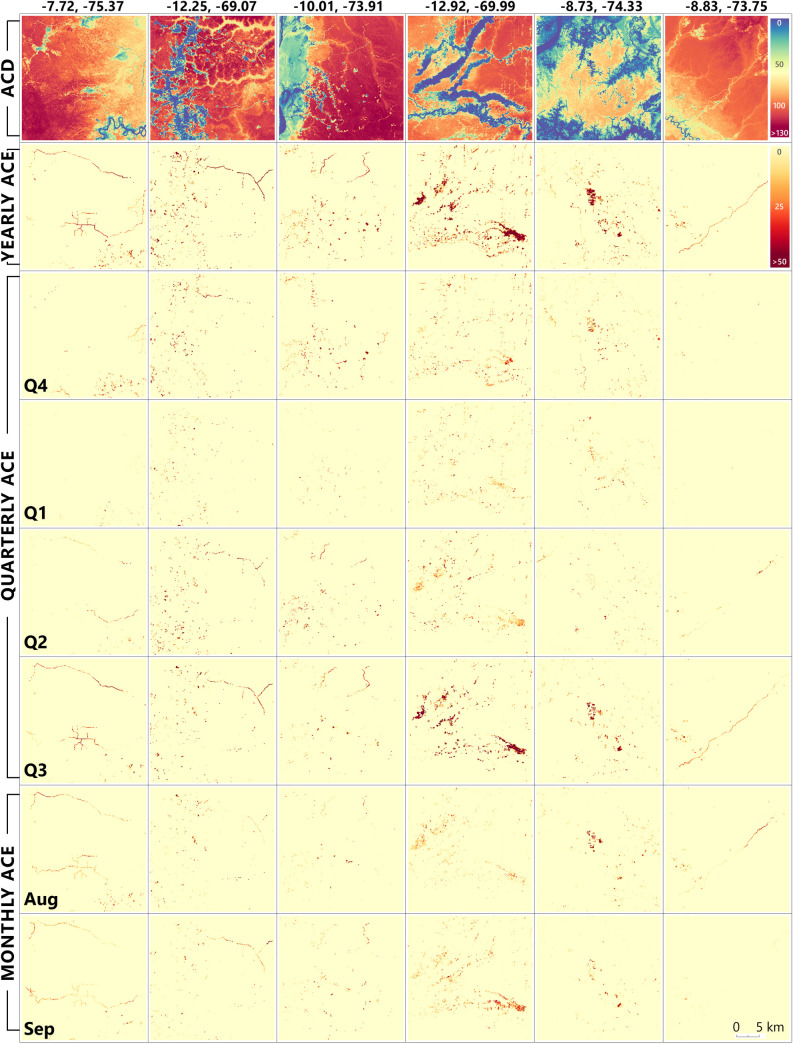
Examples of ACE tracked over time with yearly, quarterly, and monthly emissions. The first row depicts the ACD situation in Q3 of 2018, followed by the yearly, quarterly and monthly ACE. Coordinates of each scene are shown above each column.

Spatial context information (geographic coordinates) can describe broad-scale trends in the pattern of the aboveground carbon distribution [41, 50]. A good ACD estimation model should be able to predict small-scale forest changes and including the spatial context in a regression can lead to incorrectly rejecting null hypotheses of no relationship [50]. In our case, that would mean the lack of a model’s ability to identify small-scale changes, like deforestation surrounded by intact forest. However, including predictors that depict these changes (spectral, vegetation indices, and radar) overpass the importance of the spatial context and these small-scale disturbances are captured by the ACD estimations and, furthermore, present in the ACE estimations (Fig 9). An important reason for using spatial context in our modelling framework was to minimize the influence of possible seamlines from the Planet Dove and Sentinel-1 Peru-wide mosaics in the final ACD estimations.

A total aboveground carbon stock of 7.138 Pg C was estimated for 2018, a value similar to 6.922 Pg C in 2012 [34] and to 6.928 Pg C in 2017 [18], which used the 1-ha resolution for estimations. It is also similar to 6.903 Pg carbon in aboveground biomass reported in 2010 by the FAO’s Global Forest Resources Assessment (GFRA) [51], but lower than 11.564 Pg C reported by Baccini et al. [3], ~9.3 Pg C found by Saatchi et al. [52], and 9.79 Pg C reported by Avitabile et al. [53], which used lower resolution remotely sensed data. We obtained a low RMSE (20.82 Mg C ha^-1^) and high R^2^ value (0.78) for the dry season of 2018, with consistent results over all the quarterly and monthly periods analyzed. The remaining 22–25% of unexplained variance can be minimized by using additional remotely sensed predictors and image processing techniques. For example, texture analysis on high resolution Planet data [18, 54], hyperspectral data [55], or other multispectral imagery [56–58] have been successfully used for aboveground biomass and carbon estimation purposes. However, using extra predictors will require a trade-off between accuracy of the model, computational resources needed and the temporal frequency of the analysis.

Annual ACE totaled 20.08 Tg C, which is 24.3% higher than the annual ACE reported by FAO’s GFRA between 2005 and 2010 (15.2 Tg C) [51], but similar with the annual ACE rate reported by Csillik et al. [18] between 2012 and 2017 (19.2 Tg C). Interestingly, the summed quarterly ACE embedded into the annual timeline of 20.08 Tg C totaled 36.8 Tg C, an 83% increase in ACE when the analysis was carried out quarterly instead of annually. Thus, tracking gross changes in carbon stocks more closely over time generates a more detailed accounting of losses (Fig 9). Moreover, using short time intervals in the mosaicking of Planet Dove images increases the likelihood that subsequent analyses will depict the carbon-change events between observation periods. Even more, the monthly mosaics detected what is happening in a given quarter and what factors lead to an increase or decrease in the ACE (Fig 9). In our case, the monthly analysis detected 13.5 Tg C inside Q3 of 2018. The quarterly and monthly overestimations can also be partially attributable to masking these ACE estimates using the yearly Forest Loss layer from Global Forest Watch [19]. Thus, there is a need for frequent monitoring of forest change [47, 59, 60] that can be coupled with the increased frequency of ACE estimates for near-real time operational applications. Careful consideration needs to be given to estimating the uncertainties of ACE and how these propagate through the estimation process, especially over such small periods. Nevertheless, more accurate and updated canopy height measurements can help to reduce the uncertainty in ACD estimates [61] by minimizing the temporal mismatch between the input and target datasets [53].

Our high-resolution ACE monitoring approach identified biogeographically relevant targets for carbon storage management at the national scale [34], and showed the areas most threatened by land conversion processes. With only 1.963 Pg C protected in different national, regional or communal forms of protection and another 1.079 Pg C in their highly exposed buffer zones, our results are helpful in order to increase protection, enhance the management and minimize the effects of carbon emissions with near-real time analysis of ACE. With consistent results over all periods analyzed, our approach can be readily updated over time and used for monitoring purposes in Peru and other countries.

Deep learning workflows are increasingly used to uncover patterns and insights from geospatial data [62, 63]. We developed a deep learning model workflow capable of capturing complex non-linear relationships between target and input variables in a high-performance computing environment. Deep learning was previously used for aboveground biomass [36] and ACD estimation [30, 38] over smaller regions, but our deep learning model was applied on a larger area (>128.5 mil ha). An important advantage of our study is the creation of a single, nationwide model that can ingest the enormous amount of data required for high-resolution reporting in space and time.

One limitation of our study relates to the time difference between the airborne LiDAR campaigns and Planet Dove and Sentinel-1 datasets. We are aware that land cover changes occurred between 2011–2013 and 2017–2018 so an initial screening of the 6,761,624 ha of LiDAR sampling [34] was performed by removing possible inconsistencies between the timeframe analyzed, resulting in 6,176,586 ha of LiDAR samples used in our analysis. The influence of the small percentage of mismatch due to land cover changes is minimized by the vast amount of data used in the analysis, as well as using a spatial resolution of 1 ha for ACD and ACE mapping. Future studies can use current spaceborne LiDAR missions, like GEDI (Global Ecosystems Dynamics Investigator) [64], for updated large-scale analysis of ACD and ACE.

Monitoring ACE in tropical forests is of major interest in the context of climate change mitigation [4]. International agreements such as the REDD+ initiative are committed to reducing deforestation and forest degradation related to anthropogenic actions, but policy measures are still difficult and hard to implement [2]. The proposed method detects both natural and anthropogenic disturbances and further developments need to focus on better separating the types and sources of deforestation and forest degradation. Our study aimed to advance the aboveground carbon monitoring through an objective and spatially explicit methodology that brings the monitoring capabilities towards near-real time, more effective and actionable.

## Conclusions

We developed a high spatial and temporal resolution approach for estimating ACE in Peru using a deep learning workflow combined with Planet Dove, Sentinel-1 data, and airborne LiDAR. The ACE estimates were masked using the Forest Loss layer provided by the Global Forest Watch [19], thus providing enhanced results that better overlap the land-use change for each period analyzed. The provisioning of annual, quarterly and monthly estimates of ACE represents an important step towards a near-real time monitoring system for tropical aboveground carbon.

## Supporting information

S1 Fig Statistics of Planet Dove mosaics. The number of Planet Dove scenes comprising quarterly and monthly mosaics and the final cloud cover percentage of these mosaics.
(DOCX)

S2 Fig The research design in estimating aboveground carbon density (ACD) and aboveground carbon emissions (ACE). ACD was estimated quarterly and monthly, while ACE was estimated at yearly, quarterly, and monthly time periods.
(DOCX)

## References

[1] Bastin J-F, Finegold Y, Garcia C, Mollicone D, Rezende M, Routh D, et al The global tree restoration potential. Science. 2019;365: 76–79. 10.1126/science.aax0848 31273120

[2] Mitchard ETA. The tropical forest carbon cycle and climate change. Nature. 2018;559: 527–534. 10.1038/s41586-018-0300-2 30046067

[3] Baccini A, Walker W, Carvalho L, Farina M, Sulla-Menashe D, Houghton RA. Tropical forests are a net carbon source based on aboveground measurements of gain and loss. Science. 2017;358: 230–234. 10.1126/science.aam5962 28971966

[4] de Andrade RB, Balch JK, Parsons AL, Armenteras D, Roman-Cuesta RM, Bulkan J. Scenarios in tropical forest degradation: carbon stock trajectories for REDD. Carbon Balance Manag. 2017;12: 6 10.1186/s13021-017-0074-0 28413850 PMC5344878

[5] Ramankutty N, Gibbs HK, Achard F, Defries R, Foley JA, Houghton RA. Challenges to estimating carbon emissions from tropical deforestation. Glob Chang Biol. 2007;13: 51–66.

[6] Mitchard ET, Saatchi SS, Baccini A, Asner GP, Goetz SJ, Harris NL, et al Uncertainty in the spatial distribution of tropical forest biomass: a comparison of pan-tropical maps. Carbon Balance Manag. 2013;8: 10 10.1186/1750-0680-8-10 24161143 PMC4175488

[7] Nomura K, Mitchard ETA, Bowers SJ, Patenaude G. Missed carbon emissions from forests: comparing countries’ estimates submitted to UNFCCC to biophysical estimates. Environ Res Lett. 2019;14: 024015.

[8] Bos AB, De Sy V, Duchelle AE, Herold M, Martius C, Tsendbazar N-E. Global data and tools for local forest cover loss and REDD+ performance assessment: Accuracy, uncertainty, complementarity and impact. Int J Appl Earth Obs Geoinf. 2019;80: 295–311.

[9] Asner GP. Painting the world REDD: addressing scientific barriers to monitoring emissions from tropical forests. Environ Res Lett. 2011;6: 021002.

[10] De Sy V, Herold M, Achard F, Asner GP, Held A, Kellndorfer J, et al Synergies of multiple remote sensing data sources for REDD+ monitoring. Current Opinion in Environmental Sustainability. 2012;4: 696–706.

[11] Herold M, Carter S, Avitabile V, Espejo AB, Jonckheere I, Lucas R, et al The Role and Need for Space-Based Forest Biomass-Related Measurements in Environmental Management and Policy. Surv Geophys. 2019 10.1007/s10712-019-09510-6

[12] Chave J, Andalo C, Brown S, Cairns MA, Chambers JQ, Eamus D, et al Tree allometry and improved estimation of carbon stocks and balance in tropical forests. Oecologia. 2005;145: 87–99. 10.1007/s00442-005-0100-x 15971085

[13] Duncanson L, Armston J, Disney M, Avitabile V. The importance of consistent global forest aboveground biomass product validation. Surv Geophys. 2019 Available: https://link.springer.com/article/10.1007/s10712-019-09538-8 31395994 10.1007/s10712-019-09538-8 PMC6647371

[14] Quegan S, Le Toan T, Chave J, Dall J, Exbrayat J-F, Minh DHT, et al The European Space Agency BIOMASS mission: Measuring forest above-ground biomass from space. Remote Sens Environ. 2019;227: 44–60.

[15] Reiche J, Lucas R, Mitchell AL, Verbesselt J, Hoekman DH, Haarpaintner J, et al Combining satellite data for better tropical forest monitoring. Nat Clim Chang. 2016;6: 120.

[16] Fan L, Wigneron J-P, Ciais P, Chave J, Brandt M, Fensholt R, et al Satellite-observed pantropical carbon dynamics. Nat Plants. 2019 10.1038/s41477-019-0478-9 31358958

[17] IPCC. IPCC Guidelines for National Greenhouse Gas Inventories. 2006.

[18] Csillik O, Kumar P, Mascaro J, O’Shea T, Asner GP. Monitoring tropical forest carbon stocks and emissions using Planet satellite data. Sci Rep. 2019;9: 17831 10.1038/s41598-019-54386-6 31780757 PMC6882785

[19] Hansen MC, Potapov PV, Moore R, Hancher M, Turubanova SA, Tyukavina A, et al High-resolution global maps of 21st-century forest cover change. Science. 2013;342: 850–853. 10.1126/science.1244693 24233722

[20] ter Steege H, Pitman NCA, Phillips OL, Chave J, Sabatier D, Duque A, et al Continental-scale patterns of canopy tree composition and function across Amazonia. Nature. 2006;443: 444–447. 10.1038/nature05134 17006512

[21] Hansen MC, Krylov A, Tyukavina A, Potapov PV, Turubanova S, Zutta B, et al Humid tropical forest disturbance alerts using Landsat data. Environ Res Lett. 2016;11: 034008.

[22] Team Planet. Planet Application Program Interface: In Space for Life on Earth. San Francisco, CA; 2017 Available: https://api.planet.com

[23] Team Planet. Planet Imagery Product Specifications. San Francisco, CA; 2019 Available: https://learn.planet.com/rs/997-CHH-265/images/Planet_Combined_Imagery_Product_Specs_letter_screen.pdf

[24] Team Planet. Surface Reflectance Basemaps—Technical Datasheet. 2019 Available: https://learn.planet.com/rs/997-CHH-265/images/Surface%20Reflectance%20Basemaps%20Technical%20Datasheet%20March%202019.pdf

[25] Tucker CJ. Red and photographic infrared linear combinations for monitoring vegetation. Remote Sens Environ. 1979;8: 127–150.

[26] Huete A, Didan K, Miura T, Rodriguez EP, Gao X, Ferreira LG. Overview of the radiometric and biophysical performance of the MODIS vegetation indices. Remote Sens Environ. 2002;83: 195–213.

[27] Gitelson AA, Kaufman YJ, Merzlyak MN. Use of a green channel in remote sensing of global vegetation from EOS-MODIS. Remote Sens Environ. 1996;58: 289–298.

[28] Frampton WJ, Dash J, Watmough G, Milton EJ. Evaluating the capabilities of Sentinel-2 for quantitative estimation of biophysical variables in vegetation. ISPRS J Photogramm Remote Sens. 2013;82: 83–92.

[29] Torres R, Snoeij P, Geudtner D, Bibby D, Davidson M, Attema E, et al GMES Sentinel-1 mission. Remote Sens Environ. 2012;120: 9–24.

[30] Asner GP, Brodrick PG, Philipson C, Vaughn NR, Martin RE, Knapp DE, et al Mapped aboveground carbon stocks to advance forest conservation and recovery in Malaysian Borneo. Biol Conserv. 2018;217: 289–310.

[31] Gorelick N, Hancher M, Dixon M, Ilyushchenko S, Thau D, Moore R. Google Earth Engine: Planetary-scale geospatial analysis for everyone. Remote Sens Environ. 2017;202: 18–27.

[32] Veci L, Lu J, Prats-Iraola P, Scheiber R, Collard F, Fomferra N, et al The sentinel-1 toolbox. Proceedings of the IEEE International Geoscience and Remote Sensing Symposium (IGARSS). IEEE; 2014 pp. 1–3.

[33] Jarvis A, Guevara E, Reuter HI, Nelson AD. Hole-filled SRTM for the globe: version 4: data grid. 2008 [cited 10 Sep 2019]. Available: https://research.utwente.nl/en/publications/hole-filled-srtm-for-the-globe-version-4-data-grid

[34] Asner GP, Knapp DE, Martin RE, Tupayachi R, Anderson CB, Mascaro J, et al Targeted carbon conservation at national scales with high-resolution monitoring. Proceedings of the National Academy of Sciences. 2014;111: E5016–E5022. 10.1073/pnas.1419550111 25385593 PMC4250114

[35] Asner GP, Knapp DE, Boardman J, Green RO, Kennedy-Bowdoin T, Eastwood M, et al Carnegie Airborne Observatory-2: Increasing science data dimensionality via high-fidelity multi-sensor fusion. Remote Sens Environ. 2012;124: 454–465.

[36] Zhang L, Shao Z, Liu J, Cheng Q. Deep Learning Based Retrieval of Forest Aboveground Biomass from Combined LiDAR and Landsat 8 Data. Remote Sensing. 2019;11: 1459.

[37] Narine LL, Popescu SC, Malambo L. Synergy of ICESat-2 and Landsat for Mapping Forest Aboveground Biomass with Deep Learning. Remote Sensing. 2019;11: 1503.

[38] Csillik O, Asner GP. Aboveground carbon emissions from gold mining in the Peruvian Amazon. Environ Res Lett. 2020;15: 014006.

[39] LeCun Y, Bengio Y, Hinton G. Deep learning. Nature. 2015;521: 436–444. 10.1038/nature14539 26017442

[40] Kingma DP, Ba J. Adam: A Method for Stochastic Optimization. arXiv [cs.LG]. 2014 Available: http://arxiv.org/abs/1412.6980

[41] Mascaro J, Asner GP, Knapp DE, Kennedy-Bowdoin T, Martin RE, Anderson C, et al A tale of two “Forests”: Random Forest machine learning aids tropical Forest carbon mapping. PLoS One. 2014;9: 12–16. 10.1371/journal.pone.0085993 24489686 PMC3904849

[42] Shendryk Y, Rist Y, Ticehurst C, Thorburn P. Deep learning for multi-modal classification of cloud, shadow and land cover scenes in PlanetScope and Sentinel-2 imagery. ISPRS J Photogramm Remote Sens. 2019;157: 124–136.

[43] Shi Y, Huang W, Ye H, Ruan C, Xing N, Geng Y, et al Partial Least Square Discriminant Analysis Based on Normalized Two-Stage Vegetation Indices for Mapping Damage from Rice Diseases Using PlanetScope Datasets. Sensors. 2018 10.3390/s18061901 29891814 PMC6021985

[44] Pandit S, Tsuyuki S, Dube T. Estimating above-ground biomass in sub-tropical buffer zone community forests, Nepal, using Sentinel 2 data. Remote Sensing. 2018;10 10.3390/rs10040601

[45] Karlson M, Ostwald M, Reese H, Sanou J, Tankoano B, Mattsson E. Mapping Tree Canopy Cover and Aboveground Biomass in Sudano-Sahelian Woodlands Using Landsat 8 and Random Forest. Remote Sensing. 2015;7: 10017–10041.

[46] Avitabile V, Baccini A, Friedl MA, Schmullius C. Capabilities and limitations of Landsat and land cover data for aboveground woody biomass estimation of Uganda. Remote Sens Environ. 2012;117: 366–380.

[47] Reiche J, Hamunyela E, Verbesselt J, Hoekman D, Herold M. Improving near-real time deforestation monitoring in tropical dry forests by combining dense Sentinel-1 time series with Landsat and ALOS-2 PALSAR-2. Remote Sens Environ. 2018;204: 147–161.

[48] Lohberger S, Stängel M, Atwood EC, Siegert F. Spatial evaluation of Indonesia’s 2015 fire‐affected area and estimated carbon emissions using Sentinel‐1. Glob Chang Biol. 2018;24: 644–654. 10.1111/gcb.13841 28746734

[49] Castillo JAA, Apan AA, Maraseni TN, Salmo SG. Estimation and mapping of above-ground biomass of mangrove forests and their replacement land uses in the Philippines using Sentinel imagery. ISPRS J Photogramm Remote Sens. 2017;134: 70–85.

[50] Miller J, Franklin J, Aspinall R. Incorporating spatial dependence in predictive vegetation models. Ecol Modell. 2007;202: 225–242.

[51] FAO. Global Forest Resources Assessment 2015. 2015.

[52] Saatchi SS, Harris NL, Brown S, Lefsky M, Mitchard ETA, Salas W, et al Benchmark map of forest carbon stocks in tropical regions across three continents. Proc Natl Acad Sci U S A. 2011;108: 9899–9904. 10.1073/pnas.1019576108 21628575 PMC3116381

[53] Avitabile V, Herold M, Heuvelink GBM, Lewis SL, Phillips OL, Asner GP, et al An integrated pan-tropical biomass map using multiple reference datasets. Glob Chang Biol. 2016;22: 1406–1420. 10.1111/gcb.13139 26499288

[54] Csillik O, Kumar P, Asner GP. Challenges in Estimating Tropical Forest Canopy Height from Planet Dove Imagery. Remote Sensing. 2020;12: 1160.

[55] Vaglio Laurin G, Chen Q, Lindsell JA, Coomes DA, Frate FD, Guerriero L, et al Above ground biomass estimation in an African tropical forest with lidar and hyperspectral data. ISPRS J Photogramm Remote Sens. 2014;89: 49–58.

[56] Forkuor G, Benewinde Zoungrana J-B, Dimobe K, Ouattara B, Vadrevu KP, Tondoh JE. Above-ground biomass mapping in West African dryland forest using Sentinel-1 and 2 datasets—A case study. Remote Sens Environ. 2020;236: 111496.

[57] Tang X, Hutyra LR, Arévalo P, Baccini A, Woodcock CE, Olofsson P. Spatiotemporal tracking of carbon emissions and uptake using time series analysis of Landsat data: A spatially explicit carbon bookkeeping model. Sci Total Environ. 2020;720: 137409 10.1016/j.scitotenv.2020.137409 32145612

[58] Nguyen TH, Jones SD, Soto-Berelov M, Haywood A, Hislop S. Monitoring aboveground forest biomass dynamics over three decades using Landsat time-series and single-date inventory data. Int J Appl Earth Obs Geoinf. 2020;84: 101952.

[59] Woodcock CE, Loveland TR, Herold M, Bauer ME. Transitioning from change detection to monitoring with remote sensing: A paradigm shift. Remote Sens Environ. 2020;238: 111558.

[60] Finer M, Novoa S, Weisse MJ, Petersen R, Mascaro J, Souto T, et al Combating deforestation: From satellite to intervention. Science. 2018;360: 1303–1305. 10.1126/science.aat1203 29930127

[61] Vaglio Laurin G, Ding J, Disney M, Bartholomeus H, Herold M, Papale D, et al Tree height in tropical forest as measured by different ground, proximal, and remote sensing instruments, and impacts on above ground biomass estimates. Int J Appl Earth Obs Geoinf. 2019;82: 101899.

[62] Brodrick PG, Davies AB, Asner GP. Uncovering Ecological Patterns with Convolutional Neural Networks. Trends Ecol Evol. 2019;34: 734–745. 10.1016/j.tree.2019.03.006 31078331

[63] Reichstein M, Camps-Valls G, Stevens B, Jung M, Denzler J, Carvalhais N, et al Deep learning and process understanding for data-driven Earth system science. Nature. 2019;566: 195–204. 10.1038/s41586-019-0912-1 30760912

[64] Dubayah R, Blair JB, Goetz S, Fatoyinbo L, Hansen M, Healey S, et al The Global Ecosystem Dynamics Investigation: High-resolution laser ranging of the Earth’s forests and topography. Science of Remote Sensing. 2020;1: 100002.

