# Supplementary material for: Near-real time aboveground carbon emissions in Peru
Source: PLoS One. 2020 Nov 2;15(11):e0241418. doi: 10.1371/journal.pone.0241418 (PMC7605693; doi:10.1371/journal.pone.0241418)
Supplement: S1 Fig — The number of Planet Dove scenes comprising quarterly and monthly mosaics and the final cloud cover percentage of these mosaics. (DOCX) [file pone.0241418.s001.docx]

**
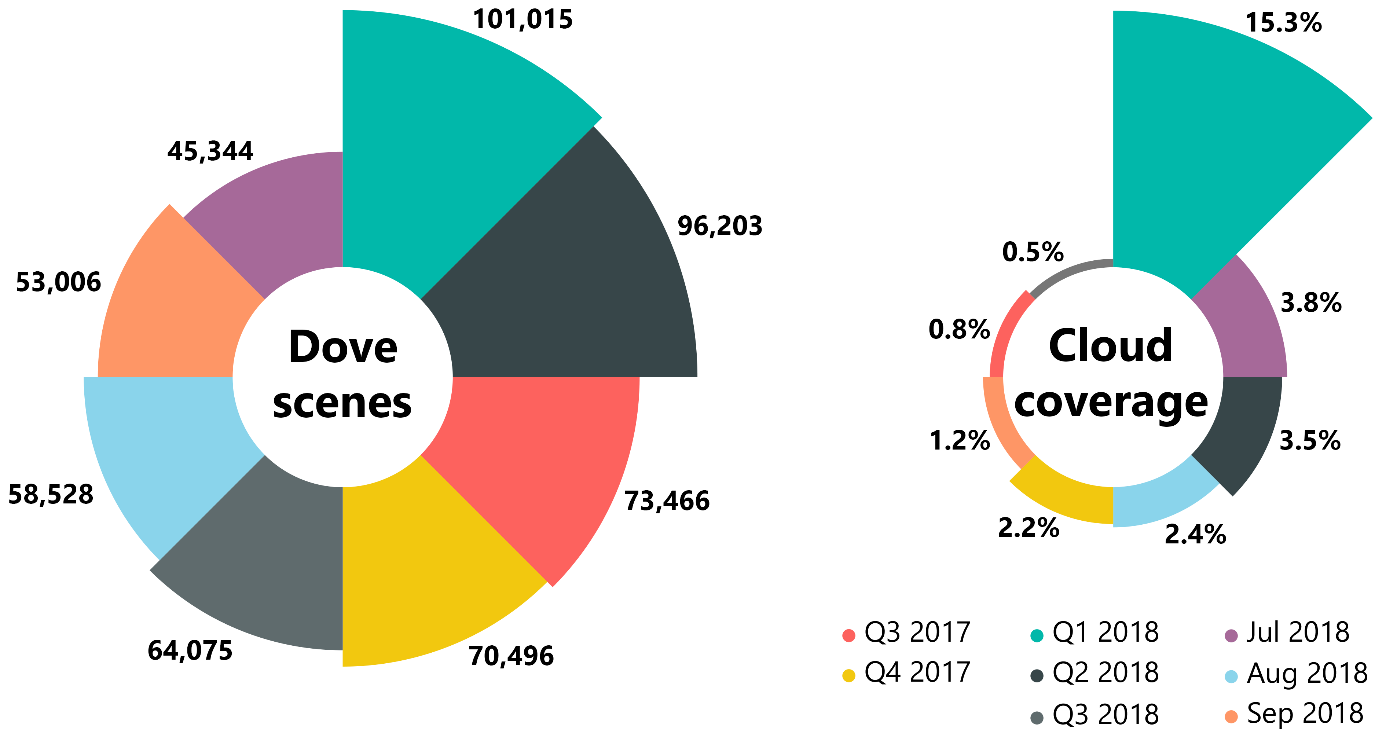
**

**S1 Fig. Statistics of Planet Dove mosaics.** The number of Planet Dove scenes comprising quarterly and monthly mosaics and the final cloud cover percentage of these mosaics.
