# Supplementary material for: Near-real time aboveground carbon emissions in Peru
Source: PLoS One. 2020 Nov 2;15(11):e0241418. doi: 10.1371/journal.pone.0241418 (PMC7605693; doi:10.1371/journal.pone.0241418)
Supplement: S2 Fig — ACD was estimated quarterly and monthly, while ACE was estimated at yearly, quarterly, and monthly time periods. (DOCX) [file pone.0241418.s002.docx]

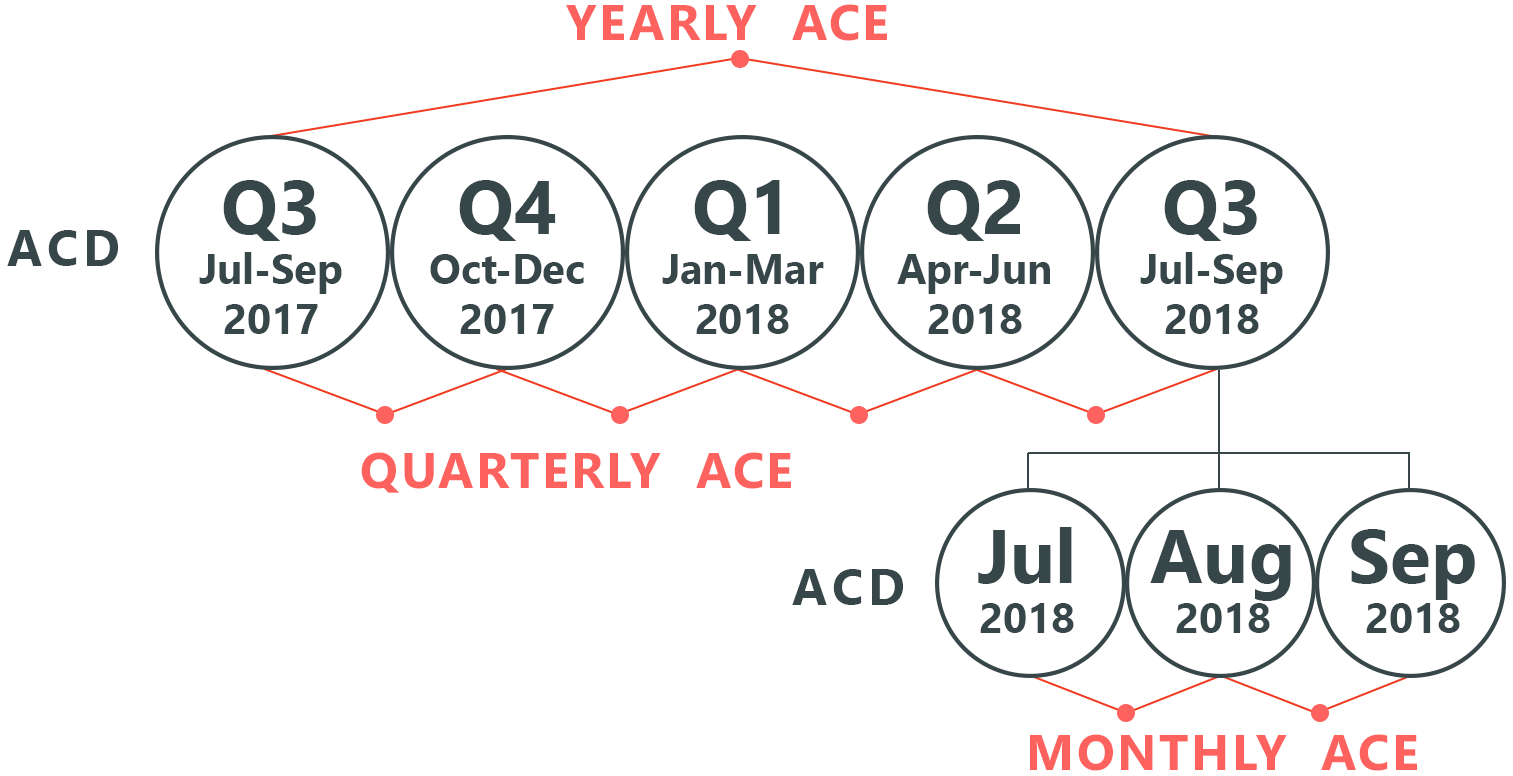


**S2 Fig.** **The research design in estimating aboveground carbon density (ACD) and aboveground carbon emissions (ACE).** ACD was estimated quarterly and monthly, while ACE was estimated at yearly, quarterly, and monthly time periods.
